# Physical Fitness Level and Mood State Changes in Basic Military Training

**DOI:** 10.3390/ijerph17239115

**Published:** 2020-12-06

**Authors:** Hyoyeon Ahn, Yongse Kim, Jaeuk Jeong, Youngho So

**Affiliations:** 1 Department of Physical Education, College of Education, Seoul National University, Seoul 08826, Korea; ahy0522@snu.ac.kr (H.A.); planmars@snu.ac.kr (J.J.); 2 College of Sports Science, Dankook University, Cheonan 31116, Korea; Soyh0920@gmail.com

**Keywords:** basic military training (BMT), physical fitness level, mood state, total mood disturbance score, latent growth model

## Abstract

The purpose of this study was to investigate the change of fitness level and mood states in the basic military training (BMT) for midshipmen using latent growth model analysis. A total of 285 midshipmen were selected as participants in BMT. The results were as follow: First, the slope of low initial fitness level increase higher than high initial fitness level. Second, there is no significant on relation between the slope of total mood disturbance score (TMD) and initial TMD level. Third, initial fitness level might increase the difference in participants’ mood state. To Sum up, participants in low initial fitness level scored lower on the results of initial TMD than people in high initial fitness level, and the rate of change in TMD of the stronger participants was larger than the others. Thus, we suggested that to consideration not only fitness level but also psychological, social aspect during in BMT.

## 1. Introduction

The basic military training (BMT) is a 5-week program for prospective cadets summoned to each armed forces’ military academy. It focuses on military knowledge such as cultivation of basic fitness, national ideology, concepts of national security, and drills, which are qualities that cadets must be equipped with. The average age range of prospective cadets that participate in the BMT is 19–22, and they are reasonably similar in body composition to those in the same age group. According to a national status survey, the average body mass index (BMI) of the 19–24 age group, which is the same age group as prospective cadets, demonstrated a rising trend from the early 2000s until recently [1]. As such, the rise of BMI among those aged 19–24 is related to the social phenomena of rising obesity rates and deteriorating fitness among youths [2,3]. Thus, prospective cadets, who are taking their first steps within the military, also have similar physical attributes to average youths in the same age group. In fact, in the case of those who applied for the Naval Academy in 2011, men had similar levels as the average of those in the same age groups, and women had higher levels than the average of those in the same age groups. It has also been reported that the fitness levels shown in fitness tests during the selection process have been gradually becoming lower than in the past [4].

As if to reflect such a phenomenon, the lifestyle within the academies, which have the goal of training elite defenders of the state, and the BMT program, which is a program for providing the cadets military knowledge, are conducted with the goal of cultivating strong fitness levels by increasing the proportion of time spent in physical training. Additionally, military fitness tests are performed using categories and methods designated by the Korea Ministry of National Defense. From 2010, the existing 1500 m running has been changed to 3000 m running. As such, training contents such as the increase of distance for double march training, and the increase of time spent in physical training, are being reflected in BMT for prospective cadets, officers, and non-commissioned officer candidates. However, due to various causes such as maladjustment to strict communal living, as well as physical and mental exhaustion from intensive education and training, some people give up entering the academies during the summons period. The occurrence of these abandonment, despite Pre-enrollment medical test, mental health-related issues are reported as a major cause of problems constantly during the BMT of the US military [5,6]. For the academies that strive to train excellent soldiers, this is a source of great disappointment.

Therefore, alongside the efforts for raising the fitness levels of prospective cadets going through BMT, efforts are needed to manage and mediate changes in their mental states. However, for youths used to a freer lifestyle, five weeks of BMT is quite a long period for them to endure and overcome. Moreover, based on the dual-mode model theory [7], referring to effects of the intensity of physical exercise on emotional changes, especially for prospective cadets that have lower than average fitness levels, the high intensity training during the summons period is highly likely to induce negative emotions.

Research on the relevance of exercise to various psychological factors such as emotion and mood is being proactively conducted through studies that investigate the relationship of psychological effects with exercise, such as confirming differences in psychological variables that depend on the conditions of physical activity such as obesity and exercise [8,9,10]. In general, physical fitness is closely related to mental health in various populations [11,12,13]. For example, it is important to pay attention to young people throughout low cost strategies for improving mental health. Physical activity is often suggested as one such approach [14]. And using a retrospective cohort design, showed that midlife physical fitness is associated with a lower risk of later-life depression [15]. Especially, in military populations, the relation between physical fitness and mental health is notably relevant because of the properties of the army environment, which were requiring than any other occupation [16].

Particularly, in Yoon’s [17] research, the psychological states of athletes, rather than of average person or students, were measured periodically, and directions for checking and preventing overtraining were proposed as well. Regarding earlier studies from outside Korea, research has shown that during BMT, groups with higher fitness test records are mentally healthier than groups with lower records [5,6]. However, these studies simply examined the differences between groups, and therefore have difficulty in providing more specific explanations. Additionally, due to the differences in social and cultural backgrounds, a substantive approach is needed for domestic research. However, currently there are no related domestic studies.

As the scales that measure emotional states related to exercise, the FS (feeling scale), the SEES (subjective exercise experience scale), and the POMS (profile of mood states) are being utilized variously in the field. Among them, the POMS comprises six factors of tension, depression, anger, fatigue, vigor, and confusion. POMS is a suitable tool often used to identify the mood of an athlete. As a pioneering psychometric tool for overtraining research, the relevance of POMS as a monitoring instrument is emphasized by many studies [18].

Domestically, it has been used effectively for repeatedly measuring and observing changes in mood states during extreme situations such as in an ultra-marathon [19], or during restricted situations such as camp training [20]. Therefore, for this study that is being done on prospective cadets undergoing BMT, POMS seems to be the most appropriate scale for evaluating mood states.

On the other hand, fitness or performance capability focuses mainly on changes or growth depending on the passage of time in the field of kinesiology research. Similarly, the Latent Growth Model analysis method enables observation that considers individual differences in the relevant changes during the same time periods. Like the *t*-test and analysis of variance, this method is effective in research that attempts to confirm changes through comparing different groups and making periodical observations. Additionally, a latent growth model considers the initial levels and rate of change, and therefore enables confirmation of variability depending on the period. Furthermore, it has an advantage of verifying various models of hypothesis established by the researcher such as a normal SEM (structural equation model) [21]. Due to these advantages of LGM (latent growth model), there have been a variety of studies on changes in BMI, physical strength, physical activity and psychological factors [22,23,24,25,26].

However, due to difficulty in data collection, domestic use is currently lacking. Therefore, it is thought that an analysis applying a Latent Growth Model is a more effective approach rather than simply analyzing the differences between groups in a study that observes changing states through periodical measurements.

As such, this study’s objectives are to repeatedly examine the fitness levels and mood states of two classes of prospective cadets in identical periods during five weeks of BMT, and then to verify what changes are happening during the period through such longitudinal data. The results derived from the study can later be used as baseline data needed for creating a scientific training program that considers physical and mental levels as well as characteristics of the prospective cadets. It can also be used as a basis for deciding whether the program is applicable to prospective cadets who have deteriorating fitness levels overall. Furthermore, there is academic significance in revealing whether the relationship between exercise and emotion have similar characteristics in terms of military training.

## 2. Method

### 2.1. Participants and Data Collection

Participants were selected from 320 prospective cadets from two classes that were summoned to the Naval Academy for two years and participated in the BMT. Data were collected throughout the five weeks of BMT via 285 participants, which excluded those who left the Academy, those who were sick, and those who did not properly respond on the survey. Three times of fitness test records and 855 pages of mood state test results were used in the study. To ensure the validity of the measurement data, every mood state test was conducted at the same time in the middle of the fitness test. The general characteristics of the participants are as shown in Table 1. All participants of this study received a full explanation of the research aims, and consent was obtained from them before administering the questionnaire. And researchers completed a research ethics education course of Collaborative Institutional Training Initiative (CITI), and the entire procedures were reviewed and approved by the Republic of Korea Naval Academy.

### 2.2. Measurement Items and Methods

#### 2.2.1. Military Fitness Test (Push-Ups, Sit-Ups, 3000 m Running)

Based on the fitness test guide from the Korea Ministry of National Defense, the military fitness test is carried out for each branch of the armed forces and consists of three tasks of push-ups, sit-ups, and 3000 m running. During the five weeks of training, the subjects were assessed three times on each item at the first, third, and fifth weeks.

The results of the fitness test were scored via assigning them into a formula that can score measurements according to the Naval Academy fitness test procedures. The highest achievable score for each event is 25 points for push-ups, 30 points for sit-ups, and 45 points for 3000 m running, with 100 as the total score. Furthermore, to increase the accuracy of measurement, the break and standby periods before measuring each event were adjusted to be identical. The scoring for each event and the conversion formula are shown in Table 2.

#### 2.2.2. Profile of Mood State (POMS)

As a scale for measuring mood states, the K-POMS-B (Korean version of Profile of Mood States-Brief) was used. The scale was the abbreviated version that was edited by McNair, Heuchert, and Shilony [27], based on the Profile of Mood State (POMS) originally developed by McNair, Heuchert, and Shilony [28]. It was then produced and validated in the Korean format by Yeun and Shin-Park [29]. K-POMS-B comprises 6 sub-factors (tension, depression, anger, vigor, fatigue, confusion), and each factor has 5 questions, with a total of 30 questions. Since K-POMS-B measures the immediate situation, the questions are concisely composed. For example, ”I’m angry”, ”I’m sad” and each question is measured on a 5 point Likert scale: “strongly disagree” is 1 and “strongly agree” is 5.

The Total Mood Disturbance score (TMD) was used for evaluation of a participant’s mood state, which was calculated by subtracting the score of one area (vigor), which is a positive emotion, from the combined score of the remaining 5 sub-factors (tension, depression, anger, fatigue, confusion), which are negative emotions [30].

To test the validity of the scale for mood states, a confirmatory factor analysis was carried out. Consequently, 6 categories that had factor loadings of 0.50 or less (tension number 1, depression number 2, anger number 4, vigor number 2, fatigue number 4, confusion number 4) were deleted, and to test the validity of the remaining 24 categories, TLI (Tucker Lewis Index), CFI (Comparative Fit Index), RMSEA (Root Mean Squared Error of Approximation) were verified (TLI = 0.871, CFI = 0.900, RMSEA = 0.067). Finally a total of 24 categories were used for analysis. Additionally, in examination via Cronbach’s α test, reliability of internal consistency showed relatively high levels (1st week 0.853, 3rd week 0.881, 5th week 0.886).

### 2.3. Data Processing Method

The data collected in this study were processed as following using PASW statistics 18.0 and AMOS 18.0 (SPSS Korea Datasolution Inc., Chicago, IL, USA). First, the confirmatory factor analysis to verify the construct validity and Cronbach’s α test to verify the reliability were conducted on the collected data. Second, descriptive statistical analysis was done to identify the overall characteristics of collected data, and correlation analysis was done to identify the correlation between each measurement variable. Third, Latent Growth Model analysis was done to confirm the fitness levels per measurement period and the conditions of mood state changes, while conditional Latent Growth Model analysis was done to confirm the relationship between mood state changes per measurement period depending on initial fitness levels. Statistical significance was configured as 0.05 for each procedure, and to assess the goodness of fit of the model, χ^2^ values, NFI, CFI, and RMSEA were used.

## 3. Results

### 3.1. Descriptive Statistical Analysis of Measured Categories

Descriptive statistics, including the mean, standard deviation, skewness, and kurtosis for each measurement category, are shown in Table 3. First, fitness levels all rapidly increased across each period for all three exercises. Push-ups increased on average by 19.46, sit-ups increased on average by 10.79, and 3000 m running decreased by 161.24 s on average. Therefore, the results show that overall fitness levels improved dramatically. In terms of psychological levels, for tension, depression, anger, fatigue, and confusion, the fifth week showed the lowest mean, indicating positive changes. However, for anger and fatigue, the third week showed the highest level.

Vigor, which is a positive emotion, showed the lowest level during the first week. It showed the highest in the third week and decreased again at the fifth week. Additionally, skewness and kurtosis in the mood states test generally appeared not to exceed the standard values.

### 3.2. Changes in Fitness Scores per Measurement Period

**Figure 1:**
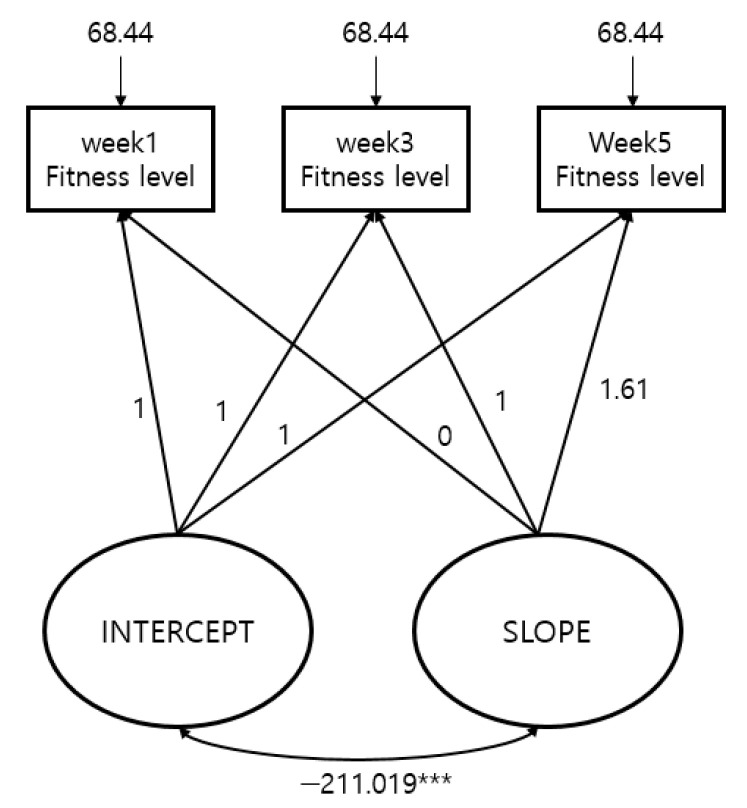
Fitness score model goodness of fit test results. *** *p* < 0.001.

The result of analysis through the Latent Growth Model to examine the changes in fitness scores per measurement period during BMT of prospective cadets are shown in Table 4 and Figure 1.

For the model’s goodness of fit test results, the RMSEA value is 0.298, but since the PCLOSE (close fit) that assumed RMSEA value of 0.05 or below selected the null hypothesis, 0.298 is statistically significant. The NFI and the CFI show the model’s goodness of fit. In addition, if the degree of freedom is small during the process of identifying the model’s goodness of fit test, RMSEA is over-estimated because it increases the likelihood of type 1 errors in RMSEA [31,32,33,34,35,36,37]. Therefore, it is more efficient to check the model’s goodness of fit in a model with a smaller degree of freedom by considering CFI and TLI that are not affected by the magnitude of the degree of freedom [35,36,38,39,40].

To observe the changes in fitness scores, the lowest score was not converted to 0 so that negative values could exist. As a result, the mean intercept was −6.308 and the slope was 33.487, which was considerably large, confirming that the fitness scores for each measurement period rapidly improved. Additionally, the variance (−211.019) between the intercept and slope of the fitness score was shown to be statistically significant (*p* < 0.001), confirming that for prospective cadets with high initial fitness levels, their slope of fitness scores increasing with time tended to decrease.

### 3.3. Changes in TMD Score per Measurement Period

**Figure 2:**
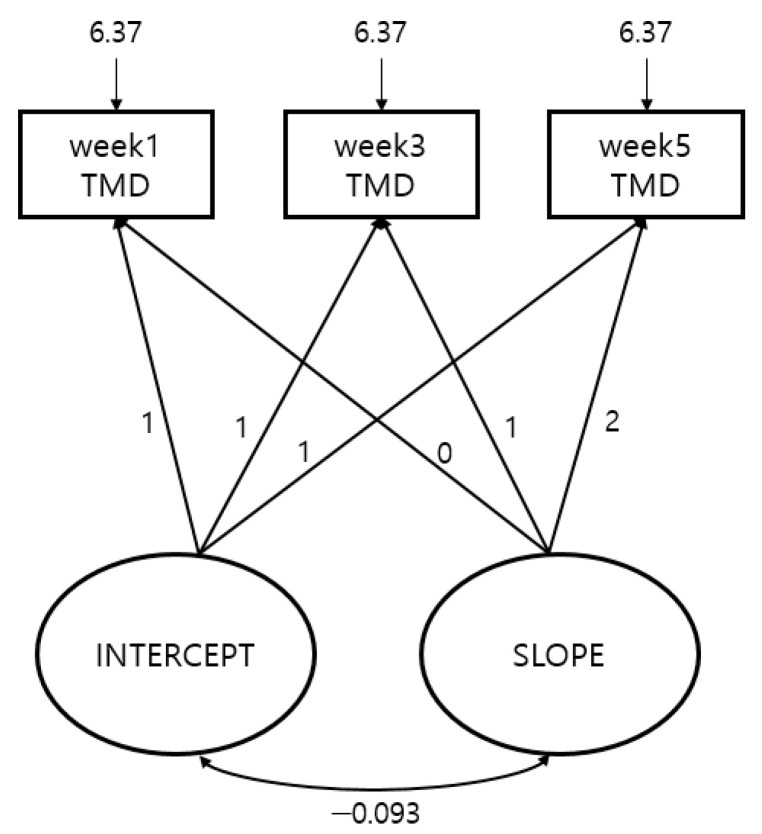
Latent growth model of TMD.

Table 5 and Figure 2 show the results of analysis through the Latent Growth Model, with the mood states per measurement period during the BMT for prospective cadets as the TMD score.

As suggested in Table 5, the model’s goodness of fit was shown as NFI = 0.966, CFI = 0.976, RMSEA = 0.090 (LO 0.034), showing that it satisfies all of the acceptance criteria for goodness of fit.

The results of analysis through the Latent Growth Model showed that the average intercept of the TMD score was 8.953, and the slope was −0.680, showing a gradual reduction as time passed. However, since the covariance (−0.093) between the intercept of the TMD score and the slope turned out to be statistically insignificant (*p* = 0.877), it was confirmed that the TMD score gradually decreased with time, with no relevance to the intercept of the TMD score. Table 5 also shows through the predicted mean value that the TMD score decreased.

### 3.4. Changes in TMD Scores per Measurement Period According to Initial Fitness Levels

**Figure 3:**
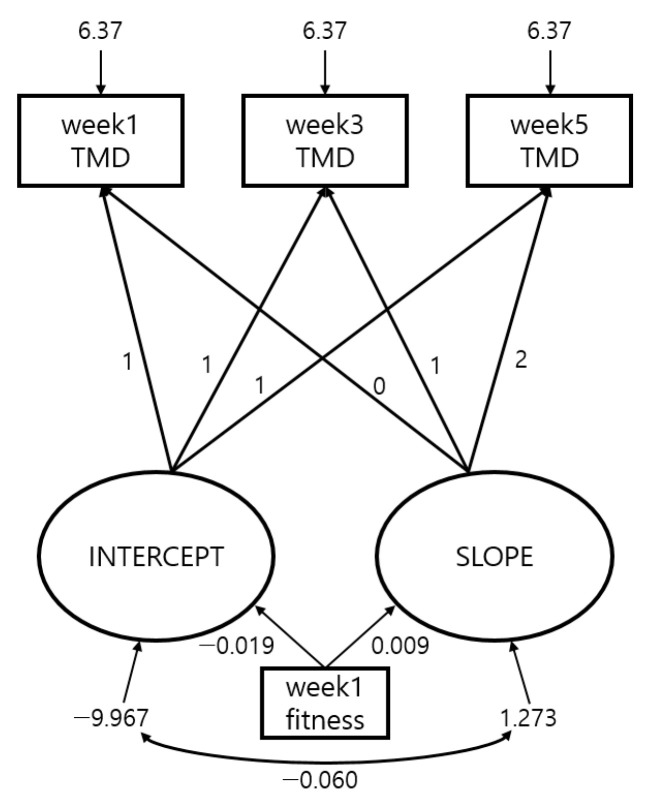
Changes in TMD score per fitness levels, model goodness of fit results.

The results as shown in Table 6 and Figure 3 were obtained via analysis through Latent Growth Model on TMD score changes per measurement period according to initial fitness levels during training of the prospective cadets.

First, the goodness of fit of the model was shown as NFI = 0.966, CFI = 0.978, RMSEA = 0.074, satisfying all of the acceptance criteria for goodness of fit.

## 4. Discussion

Existing studies that have examined the fitness and related characteristics of prospective cadets participating in BMT were limited to the goal of examining changes in physical development and fitness levels, and this was done for creating a more effective BMT program. As such, this study was carried out to offer baseline data for creating a more scientific training program for prospective cadets. Having examined the prospective cadets during BMT on their fitness levels per measurement period, changes in mood states, and changes in mood states depending on initial fitness levels, the results can be discussed as follows.

First, having examined the changes in fitness levels following measurement periods, all events showed high improvements in fitness levels. Such results are similar to the existing research results that suggested changes in fitness in the prospective cadets through BMT [41,42,43], confirming that fitness levels dramatically improved. In particular, the change in fitness levels between week one and week three was greater than the change between week three and week five. Additionally, depending on the passage of time, the rate of change for those with high initial fitness levels decreased, whereas the rate of change for those with low initial fitness levels increased. In a study with two classes that showed similar tendencies, this study differentiated itself from existing research by suggesting results from comparing differences in rate of change depending on initial fitness levels.

Next, having examined the changes in mood states per measurement period, it was shown that some negative factors (fatigue and anger) increased at the week three. However, it was confirmed that TMD scores decreased overall without any relevance to initial mood states. Such results are in accordance with Kim et al.’s [20] research, which examined the changes of mood states in female soccer players following a period of training camp, and Kang and Lee’s [19] research, which showed changes in mood state profiles of those running in 100 km ultra-marathons. Thus, the study is highly significant in that it yielded identical results to existing research that examined changes in mood state with the same measurement tools after short periods of excessive training. However, unlike changes in fitness levels, it also showed that there was an insignificant correlation between the initial mood state and the rate of change. This implies that the variable of five weeks of dynamic BMT can give rise to various changes depending on individual characteristics. Furthermore, the study is significant in that it dealt with the psychological states of prospective cadets during BMT, which was lacking in domestic research.

Lastly, the effect of initial fitness levels of prospective cadets during BMT on their mood state changes was verified through the Latent Growth Model. Such an approach was conducted under the assumption that the intensity from the BMT program felt by prospective cadets with lower fitness levels would be different. The results confirmed that on average, participants with low fitness levels had lower initial mood states, and that higher fitness levels were correlated with larger changes in mood states. Such results show that the fact that gaps in mood states can happen depending on initial fitness levels must be considered. Moreover, unlike existing studies, this study conducted the verification through a Latent Growth Model, confirming the results that the rate of change in mood state was different depending on initial fitness levels.

Particularly, participants with low initial fitness showed a highly increasing rate of change compared to participants with high initial fitness. This is a result that reflects well the nature of the goal of BMT, which is to prepare the prospective cadets for the lifestyle at the Academy. Additionally, in Shannon’s [6] research, it was shown that during BMT, the group with higher fitness test scores was mentally healthier than the group with lower fitness test scores. In Crowley et al.’s [5] research, it was shown that the group with an above average fitness test scores had 60% lower symptoms of depression than the below average group. As such, it was confirmed that the results were shown to be identical to the existing studies with similar contexts. It seems that this study has contributed toward explaining such phenomena by providing more concrete data.

## 5. Conclusions

The participants selected for this study were prospective cadets undergoing five weeks of BMT after being summoned to the Academy. After analyzing with the Latent Growth Model the effects that fitness levels per measurement period, changes in mood states, and initial fitness levels have on changes in mood state per measurement period, the following conclusions were derived.

First, during BMT, the fitness levels of the prospective cadets increased rapidly, and the rate of change was much larger for those that had lower initial fitness levels than those with higher initial fitness levels. Second, during BMT, the prospective cadets showed a tendency for positive changes in mood states. However, initial mood states and rate of change per measurement period did not show a significant relationship. Third, during BMT, prospective cadets with low initial fitness levels showed negative change tendencies in initial mood states. Those with high initial fitness levels showed a high rate of change for mood states. Furthermore, depending on the initial fitness level, there were large differences for mood states.

Such results show that BMT has positive effects on fitness levels and mood state changes in prospective cadets. Additionally, this implies that the rate of change for mood states differs by initial fitness levels. Therefore, we expect that this study’s results will be used as baseline data that will provide a foundation for creating a more effective BMT program for prospective cadets, preventing the loss of human capital and helping to train prospective cadets with healthier psychological states. Furthermore, this study’s results have been confirmed through analysis via the Latent Growth Model. Therefore, this study is significant because the results have more depth compared to existing research that examined fitness and mood states through simple group comparisons. However, there are limitations in this study in that there was no distinction between classes and genders. This study focuses on psychological changes caused by physical fitness tests among the BMT of prospective cadet. Therefore, it is determined that the psychological changes caused by prospective cadet recognizing the burden of physical fitness tests would be the same. Thus, there is a limitation to the research in that there is no distinction between classes and genders of the prospective cadet. Furthermore, the study participants are likely to be exposed to authoritative situations due to the Characteristics of the participants. Future studies need to take into account the various current situations of participants. As well as that the BMT conducted in another year showed a total difference of four hours for physical training. To examine more specific relationships on effects, later studies must verify fitness levels and changes in mood states through complex models of Latent Growth. Furthermore, it is hoped that there will be a proliferation of studies that consider various psychological and social characteristics together rather than being limited to the physical aspects of prospective cadets.

## Figures and Tables

**Table 1:** Characteristics of participants.

	Male	Female
*N*	270	15
Age	18.83 (±0.76)	18.18 (±0.54)
BMI	21.95	21.65

**Table 2:** Scoring and conversion formula for military fitness test.

Test Event	Conversion Formula		Score
Push-ups	M	(record-45) × 0.5 + 2.5	25
	F	(record-21) × 0.6 + 2.5	
Sit-ups	M	(record-52) × 0.7 + 3.0	30
	F	(record-33) × 0.6 + 3.0	
3000 m running	M	(845-record) × 0.23 + 4.5	45
	F	(980-record) × 0.21 + 4.5	

**Table 3:** Descriptive statistics for each measurement category.

Variable		Mean	SD	Skewness	Kurtosis
Push-ups	1st	42.75	0.823		
	3rd	52.50	0.992		
	5th	62.21	0.933		
Sit-ups	1st	52.52	0.841		
	3rd	59.53	0.832		
	5th	63.31	0.741		
3000 m Running	1st	936.09	7.170		
	3rd	834.44	5.433		
	5th	774.85	4.734		
Tension	1st	2.461	0.048	0.101	−0.470
	3rd	2.239	0.053	0.365	−0.487
	5th	2.068	0.054	0.603	−0.306
Depression	1st	2.108	0.053	0.587	−0.434
	3rd	2.060	0.055	0.583	−0.593
	5th	1.886	0.053	0.827	−0.235
Anger	1st	2.055	0.046	0.529	−0.325
	3rd	2.131	0.052	0.487	−0.445
	5th	2.000	0.048	0.544	−0.431
Vigor	1st	2.813	0.046	0.310	−0.107
	3rd	2.921	0.055	0.265	−0.252
	5th	2.833	0.058	0.494	0.323
Fatigue	1st	2.587	0.046	0.218	−0.108
	3rd	2.695	0.052	0.238	−0.310
	5th	2.549	0.055	0.261	−0.419
Confusion	1st	2.553	0.048	0.165	−0.429
	3rd	2.081	0.050	0.695	0.113
	5th	1.934	0.048	0.732	−0.061

**Table 4:** Changes in fitness scores per measurement period.

	Estimate	S.E.		C.R.		*p*
Intercept	−6.308	1.762		−3.580		<0.001
Slope	33.487	0.930		36.001		<0.001
Implied means	week1		week3		week5	
	−6.308		27.179		47.575	
Model fit	χ^2^ (*df*)			55.275 (2)		
	NFI			0.926		
	CFI			0.928		
	RMSEA			0.298		

**Table 5:** Latent growth model analysis of changes in POMS.

	Estimate	S.E.			C.R.		*p*
Intercept	8.953	0.234			38.230		<0.001
Slope	−0.680	0.126			−5.386		<0.001
Implied means	week 1		week 3			week 5	
	8.953		8.273			7.593	
Model fit	x^2^ (*df*)			10.238 (3)			
	NFI			0.966			
	CFI			0.976			
	RMSEA			0.090			

**Table 6:** Changes in TMD over time per fitness levels.

	Estimate	S.E.			C.R.		*p*
Intercept	8.838	0.237			37.328		<0.001
Slope	−0.623	0.128			−4.875		<0.001
Implied means	week 1		week 3			week 5	
	8.953		8.273			7.593	
Model fit	x^2^ (*df*)			10.488 (4)			
	NFI			0.966			
	CFI			0.978			
	RMSEA			0.074			
